# The time–intensity uncertainty principle in vision

**DOI:** 10.3758/s13414-026-03249-0

**Published:** 2026-04-14

**Authors:** Robert C. G. Johansson, Karin M. Bausenhart, Rolf Ulrich, Paul Kelber

**Affiliations:** Department of Psychology, University of Tübingen, Schleichstraße 4, Tübingen, 72076 Germany

**Keywords:** Uncertainty principle, Sensory processing, Time perception, Brightness perception, Visual psychophysics

## Abstract

The relationship between time perception and brightness perception remains poorly understood. Here we present a computational account linking the two domains, grounded in established principles of neural information processing in visual cortex. A nonlinear transducer maps luminance to population spike rate, while correlated gain fluctuations impose an upper bound on achievable signal-to-noise ratios. Perceptual magnitudes in both domains are decoded from the same spike-count statistics, yielding a reciprocal trade-off in perceptual resolution: brighter stimuli improve temporal precision but impair brightness sensitivity, whereas longer stimuli enhance brightness sensitivity but degrade temporal resolution. We tested this conjectured trade-off in two psychophysical experiments manipulating stimulus duration and luminance. Model predictions closely matched the behavioral data, revealing a fundamental coding limit in vision: the time–intensity uncertainty principle. This limit provides a unified explanation for near-miss relations to Weber’s law for time perception and intensity perception, Bloch-like temporal summation effects governing brightness discrimination sensitivity, and luminance-dependent shifts in duration discrimination sensitivity.

“Whenever one considers living organisms as physical or chemical systems, they must necessarily behave as such.”

— Werner (Heisenberg, 1958/2006)

One century ago, Heisenberg (1927) made one of the most groundbreaking discoveries in modern science: certain pairs of physical properties (e.g., position and momentum) are complementary and cannot be determined simultaneously with arbitrary precision. Later, Gabor (1946) demonstrated that analogous limits arise in mathematical signal processing, where the temporal and spectral resolution of signals trade off. As illustrated in Fig. 1, signals tightly localized in time necessarily occupy a broad range of frequencies, whereas longer signals occupy a narrow frequency range at the cost of temporal smearing.

Drawing inspiration from these foundational limits in physics and engineering, we propose an analogous *uncertainty principle* governing the visual perception of duration and intensity. Remarkably, this perceptual reciprocity has been formerly overlooked, and the nature of resolution dependencies between time perception and brightness perception remains unexplored. The present study addresses this gap. First, we develop a neurophysiologically grounded model of sensory coding in V1 that jointly accounts for duration and brightness discrimination thresholds. Second, we derive formal predictions showing that this integrated model entails a reciprocal trade-off between duration and brightness discrimination sensitivity. Third, we empirically validate these predictions using behavioral thresholds measured across a wide range of stimulus durations and luminance levels.

**Fig. 1:**
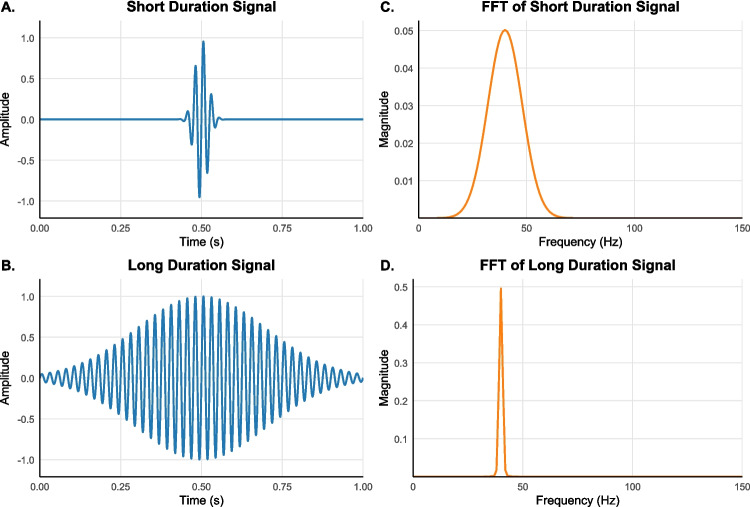
Time-domain representations of a short-duration signal (Panel **A**) and a long-duration signal (Panel **B**) embedded in Gaussian envelopes, along with their corresponding fast Fourier transforms (FFT) in the frequency domain (Panels **C** and **D**, respectively). Longer-duration signals are associated with sharper spectral peaks in the frequency domain, whereas shorter-duration signals exhibit broader frequency spreads due to the Gabor limit

## Neural coding of brightness and time

The visual system is thought to encode luminance contrast and stimulus duration through two complementary mechanisms: *rate coding*, in which stimulus intensity is conveyed by instantaneous firing rate (Diamond & Copenhagen, 1995; Albrecht & Hamilton, 1982; Sclar et al., 1990; Boynton et al., 1996; Chirimuuta & Tolhurst, 2005), and *temporal summation*, in which duration is estimated by integrating spikes over time (Gibbon, 1992; Rammsayer & Ulrich, 2001; Treisman, 1963; Simen et al., 2013). While early models of time perception invoked a centralized, supramodal timing mechanism, contemporary neurophysiological perspectives suggest that timing can emerge from intrinsic dynamics within sensory-specific cortices, without a dedicated central timer (Coull et al., 2011; Paton & Buonomano, 2018; Buhusi & Meck, 2005). This view implies that the timing of visual intervals might be computed within the visual cortex itself.

Accordingly, rate coding and temporal summation can thus be understood as distinct computations over the same sensory input: the train of spikes generated in response to a stimulus. When stimulus duration is fixed, variations in spike count reflect changes in firing rate; when rate is fixed, differences in spike count reflect changes in duration. This dual dependence motivates a class of counting models, in which perceptual estimates are based on the total number of spikes accumulated over time. Early versions of this idea were proposed in classic psychophysical accounts of both intensity discrimination (Siebert, 1965; McGill, 1967; Raab & Goldberg, 1975; Zwislocki & Jordan, 1986; Green & Luce, 1974) and duration discrimination (Treisman, 1963; Creelman, 1962; Getty, 1975; Gibbon, 1992). In this regard, meaningfully related theoretical frameworks have been invoked to explain key behavioral benchmarks in both time perception and intensity perception research.

**Fig. 2:**
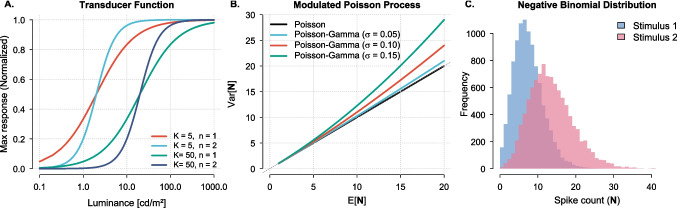
(**A**) Normalized response rates for the Naka–Rushton transducer function. The midpoint of the function increases with *K*, while the steepness increases with *n*. (**B**) Variance–mean scaling for Poisson and compound Poisson–gamma spiking. The compound process shows overdispersion, with Fano factors increasing for larger $\sigma$. (**C**) Spike-count histograms for a dim/brief (Stimulus 1; *blue*) and bright/long (Stimulus 2; *red*) visual event, illustrating a rightward shift and broadening with increased luminance or duration

## A unified model of perceptual discrimination

We formalized the link between time and brightness perception in a population-coding model in which both percepts are decoded from the same spike-count statistics in early visual cortex. The model assumes a population of *m* luminance-sensitive neurons, each generating a steady-state Poisson spike train with rate $r_i(L)$ determined by a Naka–Rushton contrast-response function (Evans et al., 1993). Under the simplifying assumption of identical contrast sensitivity across neurons, the summed population firing rate $\lambda (L)$ becomes

1 $$\begin{aligned} \lambda (L) = \sum _{i=1}^m r_i(L) = m \cdot \frac{r_{\text {max}} L^n}{L^n + K^n} = \frac{\lambda _{\text {max}} L^n}{L^n + K^n}, \end{aligned}$$

where $\lambda _{\text {max}} = m \cdot r_{\text {max}}$ is the maximum population firing rate, *K* is the semi-saturation constant, and *n* is an exponent (Fig. 2A). Although real populations vary in contrast tuning and gain, the homogeneous approximation yields an analytically tractable population response while preserving the canonical Naka–Rushton shape (scaled by *m*). The relationship between V1 contrast responses and psychophysical performance has frequently been described using population coding models of this type, sometimes incorporating heterogeneous tunings (Chirimuuta & Tolhurst, 2005; Boynton et al., 1999; Billock & Tsou, 2011; May & Zhaoping, 2011; May & Solomon, 2015a, b).

To account for cortical spike variability exceeding that predicted by a Poisson process, we introduced a random multiplicative gain factor $\textbf{g}$, applied globally and identically across all *m* channels. That is, the gain is shared across neurons, with pairwise correlations $\rho (\textbf{g}_i, \textbf{g}_j) = 1$ for all $i, j \in {1, \dots , m}$. This captures global fluctuations in population excitability, consistent with the idea that cognitive and physiological factors such as attention, arousal, and fatigue modulate neural gain across the cortex (Treisman, 1964; Goris et al., 2014; May & Solomon, 2015a; Rabinowitz et al., 2015; Ecker et al., 2014). Following previous work (Goris et al., 2014; May & Solomon, 2015a; Goris et al., 2018; May & Solomon, 2015b), we sampled $\textbf{g}$ from a gamma distribution with unit mean and variance $\sigma ^2$. This yields a doubly stochastic Poisson–gamma process in which the population spike count $\textbf{N}$ over an interval of duration *t* follows the negative binomial distribution

2 $$\begin{aligned} \textbf{N} \sim \text {NB}\left( \frac{\lambda _{\text {max}} L^n t}{L^n + K^n},\ \frac{\lambda _{\text {max}} L^n t}{L^n + K^n} + \sigma ^2 \frac{ (\lambda _{\text {max}} L^n t)^2}{(L^n + K^n)^2} \right) \end{aligned}$$

expressed here in terms of expected value and variance, the latter determined by both Poisson variability and the added gain variance (Fig. 2C).

## Deriving perceptual thresholds

We define the Weber fraction W as the smallest proportional change in the neural response that is just perceptible. Following Ulrich et al. (2023), we compute W from the coefficient of variation of $\textbf{N}$, which corresponds to a constant point on the psychometric function. For the negative binomial spike count distribution,

3 $$\begin{aligned} \text {W} = \frac{ \sqrt{\textrm{Var}[\textbf{N}]}}{\textrm{E}[\textbf{N}]} = \sqrt{\frac{L^n + K^n}{\lambda _{\text {max}} L^n t} + \sigma ^2}. \end{aligned}$$

To express sensitivity in physical stimulus units, we define the difference limen (DL) as the smallest detectable change in stimulus magnitude. For duration, the spike count scales linearly with *t*, so the duration threshold $\text {DL}_t$ (the smallest change in duration that yields a just-noticeable difference in spike count) is given by

4 $$\begin{aligned} \text {DL}_t = \text {W} \cdot t = \sqrt{t \frac{L^n + K^n}{\lambda _{\text {max}} L^n} + \sigma ^2 t^2}. \end{aligned}$$

For the luminance threshold $\text {DL}_L$, we must account for the nonlinearity of the Naka–Rushton transducer function. Using a first-order Taylor series approximation based on the local transducer slope yields

5 $$\begin{aligned} \text {DL}_L \approx \text {W} \cdot \lambda (L) \cdot \left( \frac{\partial \lambda (L)}{\partial L}\right) ^{-1} = \frac{L(L^n+K^n)\sqrt{\frac{(L^n +K^n)}{\lambda _{\text {max}}tL^{n}}+\sigma ^2}}{nK^n}. \end{aligned}$$

While this linearized form is simple and intuitive, for all model fitting we compute $\text {DL}_L$ exactly by inverting the Naka–Rushton function at the upper and lower threshold spike rates, capturing the full nonlinearity of the transducer. The inversion procedure is described in detail in Appendix A.

**Fig. 3:**
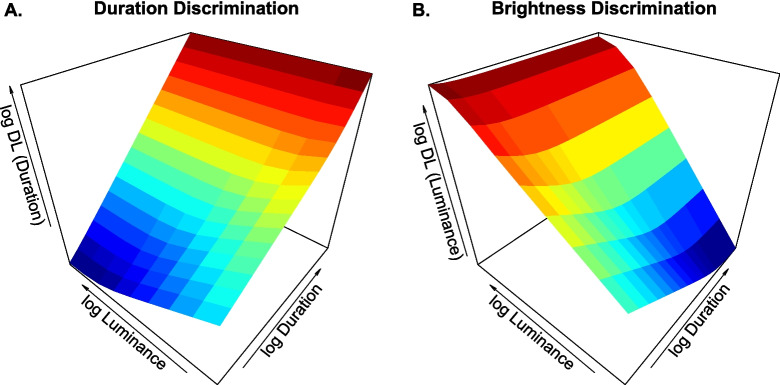
Difference limens (DL) as a function of luminance and stimulus duration, conveyed separately for duration discrimination (Panel **A**) and brightness discrimination (Panel **B**). Results are depicted assuming a Naka–Rushton transducer function and multiplicative gamma relay noise for the population spike rate $\lambda$ (see Appendix A for details)

Predictions for both DLs are shown in Fig. 3. Visual inspection of the DL gradients reveals a characteristic trade-off between time and brightness resolution: $\text {DL}_t$ increases with stimulus duration but decreases with luminance, whereas $\text {DL}_L$ increases with luminance but decreases with duration. In simple terms, one cannot simultaneously maximize visual sensitivity to both time and brightness. Some intuition for why this occurs is provided in the accompanying boxed inset. The two experiments described next provide an empirical test of our proposed time–intensity uncertainty principle in vision.
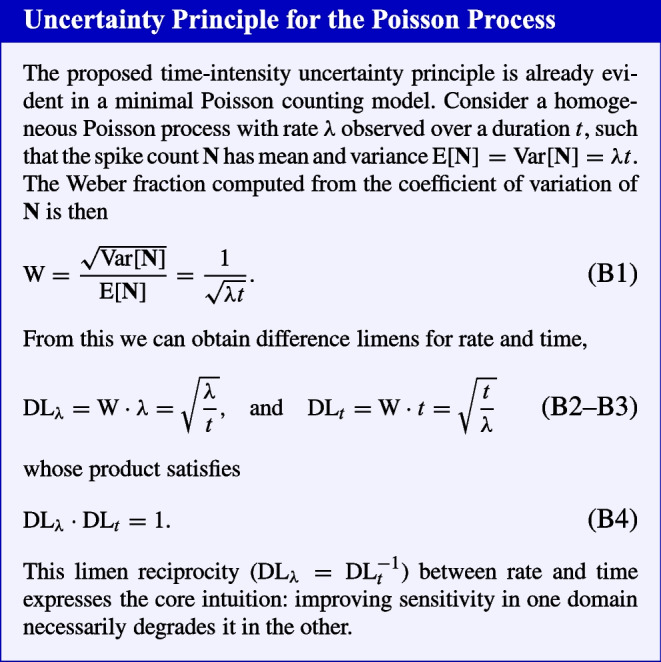


## Methods

### Participants

Two separate samples of 20 students from the University of Tübingen participated in Experiment 1 (13 female; 17 right-handed; mean age: 25 years; age range: 19–34 years) and Experiment 2 (11 female; 16 right-handed; mean age: 27 years; age range: 21–41 years). All reported normal or corrected-to-normal vision, provided informed consent, and received either course credit or 36€ for completing two sessions, each lasting 1–1.5 h. The target sample size was determined based on available testing resources.

### Procedure

The DLs were measured in a two-interval forced-choice reminder task using the weighted adaptive-staircase method (Kaernbach, 1991) targeting the points $X_{.25}$ and $X_{.75}$ of the psychometric function in each condition. Each session consisted of 800 trials, divided into 16 blocks of 50 trials. Participants completed 50 trials for each of 16 randomly interleaved staircases (eight standard stimuli $\times$ two runs). A session also comprised a 16-trial introductory practice block. The stimulus dimension to be discriminated (brightness or duration) varied between the two sessions, with session order counterbalanced across participants. Sessions were conducted on separate days at similar times in a dark testing chamber. Each trial started with the standard stimulus, followed by an empty inter-stimulus interval (1000 ms), the comparison stimulus, an empty response-terminated interval, and an empty inter-trial interval (2000 ms). No error feedback was given.

### Stimuli and apparatus

Stimulus presentation and response recording were controlled by custom Python scripts using PsychoPy (Peirce et al., 2019) and running on a Fujitsu Esprimo P910 E85+ PC (Windows 10, 64-bit). The stimuli consisted of light flashes emitted by a red photodiode (peak wavelength: 620 nm) subtending a visual angle of 0.5 degrees. The apparatus was enclosed in a black box, leaving only the photodiode visible to participants. Participants viewed the photodiode from a fixed distance of 57 cm, maintained using a chinrest. In Experiment 1, the duration and intensity of the visual stimuli were controlled using on-board pulse-width modulation (PWM) from an Arduino Uno microcontroller. The PWM signal was passed through a carbon film resistor to modulate the output voltage. Stimulus control and response recording for Experiment 2 were similar, but an external 12-bit PWM (Model: PCA9685) was used to provide finer control of luminance range. The accuracy and precision of stimulus durations were verified to sub-millisecond resolution using a Black Box Toolkit v2 (Plant et al., 2004). Luminance was calibrated with a Gigahertz Optik P-9801-TF photometer. Responses were recorded using a custom-built button box, with the left button corresponding to “first stimulus longer/brighter” and the right button to “second stimulus longer/brighter.”

### Experiment 1

Eight standard stimulus durations were used (10, 20, 40, 80, 160, 320, 640, and 1280 ms) and the standard stimulus intensity was always 3.3 cd/m$^2$. In duration discrimination, the comparison durations started at 0.6 (lower run) or 1.4 (upper run) times the standard duration (i.e., 6 and 14 ms for 10-ms standard duration, 12 and 28 ms for 20-ms standard duration, etc.), and changed with a single step size of 1/40 of the standard duration (i.e., 0.25 ms for 10-ms standard duration, 0.5 ms for 20-ms standard duration, etc.). In brightness discrimination, the comparison intensities started at 1.7 cd/m$^2$ (lower run) and 4.9 cd/m$^2$ (upper run), and changed with a single step size of 0.1 cd/m$^2$. The standard stimulus was identical in both tasks.

### Experiment 2

In both experimental tasks, eight standard stimulus intensities were used (0.04, 0.07, 0.13, 0.24, 0.47, 0.92, 1.82, and 3.63 cd/m$^2$). The standard stimulus always had a duration of 240 ms. In brightness discrimination, comparison intensities started at 0.5 and 1.5 times the standard intensity and changed with a step size of 1/25 of the standard intensity, while in duration discrimination, comparison durations started at 192 ms (lower run) and 288 ms (upper run) and changed with a single step size of 16 ms. The standard stimulus was again identical in both tasks.

### Data analysis

Difference limens (DLs) were computed for each participant by averaging the stimulus values at all reversal points in the adaptive staircases. Following Duncan Luce and Galanter (1963), empirical DLs were defined as half the difference between the means of the reversals in the upper ($X_{.75}$) and lower ($X_{.25}$) staircase: $\text {DL} = (X_{.75}- X_{.25})/2$. The DL estimates were then entered into within-subjects one-way ANOVAs to assess the effects of standard duration (Experiment 1) and standard luminance (Experiment 2) on discrimination sensitivity. Duration and brightness discrimination data were analyzed separately. Within-subject 95% confidence intervals (CIs) were computed for graphical presentation using the method described by Morey (2008).

The Naka–Rushton model with correlated gain fluctuations was fitted jointly to the brightness and duration DLs by estimating four free parameters characterizing the cortical population spike count: maximum firing rate $\lambda _{\text {max}}$, semi-saturation constant *K*, response exponent *n*, and gain variance $\sigma ^2$. Parameter estimation was performed by minimizing a weighted sum of squared residuals ($\text {SSR}_{\text {w}}$), defined as $\text {SSR}_{\text {w}} = \sum \frac{(E - O)^2}{\textrm{Var}(O)}$, where *E* and *O* denote the expected and observed DLs, respectively, and $\textrm{Var}(O)$ is the within-subject variance of the observed DLs. The optimization employed a downhill simplex algorithm. Model–data correspondence was assessed in terms of Pearson correlation tests.

Model CIs were estimated via a two-stage bootstrap procedure: First, 10,000 synthetic datasets were generated for each experiment by nonparametric resampling with replacement at the participant level. Second, the full model was re-fitted to each resampled dataset, and 95% CIs for the model were derived from the empirical distribution of these bootstrapped model predictions.

**Fig. 4:**
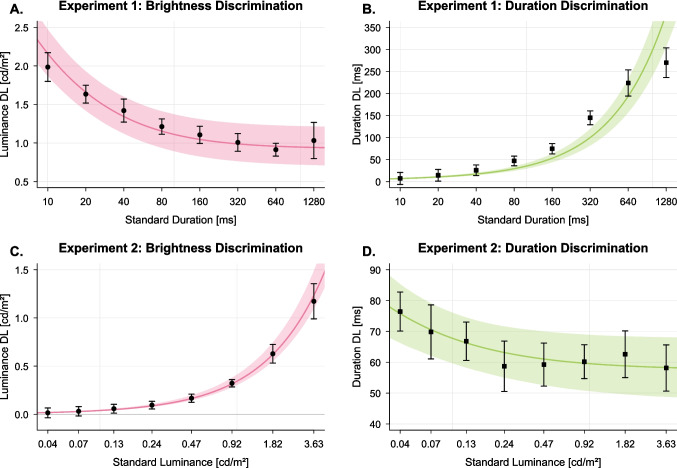
Difference limens (DLs) for brightness discrimination (A, C) and duration discrimination (B, D). *Upper panels* (**A**, **B**) show results from Experiment 1 as a function of stimulus duration; *lower panels* (**C**–**D**) show results from Experiment 2 as a function of stimulus luminance. *Error bars* indicate within-subjects 95% confidence intervals (CIs) following Morey (2008). *Shaded regions* represent 95% CIs of the model predictions based on 10,000 bootstrap-resampled datasets

**Table 1:** Parameter estimates and model fits for Experiments 1 and 2. All units are dimensionless except for $\lambda _\text {max}$ which is in Hz

Parameter	Description	Experiment 1	Experiment 2
$\lambda _\text {max}$	Maximum rate	$5.02 \times 10^4$	$1.48 \times 10^{5}$
*K*	Saturation constant	$4.80 \times 10^2$	$1.00 \times 10^{3}$
*n*	Transducer exponent	1.06	0.73
$\sigma ^2$	Relay noise	$8.58 \times 10^{-2}$	$5.73 \times 10^{-2}$
$\text {SSR}_{\text {w}}$	Goodness of fit	6.31	0.19

## Results and discussion

### Uncertainty principle depends on duration

Experiment 1 investigated how differential visual sensitivity to duration and luminance contrast depends on stimulus exposure time. Twenty participants viewed pairs of brief visual flashes, ranging from 10 to 1280 ms in duration, and judged either which flash was brighter or which lasted longer. These tasks were administered in separate sessions. On each trial, a standard flash with fixed luminance (3.31 cd/m$^2$) was paired with a comparison flash that differed either in intensity or duration.

We observed a clear reciprocal relationship between temporal resolution and intensity resolution. As stimulus duration increased, participants became progressively less accurate at detecting differences in duration but simultaneously better at judging differences in luminance. Brightness discrimination thresholds decreased over the tested range, from 1.99 cd/m$^2$ for the shortest flashes (10 ms) to 1.03 cd/m$^2$ for the longest flashes (1280 ms), $F(3.19,\ 60.61) = 28.17$, $p <.001$, $\eta _G^2 =.22$ (Greenhouse–Geisser corrected, $\epsilon =.45$). Duration discrimination thresholds increased steeply across the same range, from 7 ms to 270 ms, $F(2.10,\ 39.90) = 117.21$, $p <.001$, $\eta _G^2 =.80$, $\epsilon =.30$. Thus, gains in contrast sensitivity came at the expense of temporal sensitivity, consistent with the predicted time-–intensity trade-off.

The compound Poisson-gamma spike-count model with a Naka–Rushton transducer function adequately captured the differential sensitivity functions for both brightness discrimination, $\rho$ = .99, 95% CI = [.93, .997], $p <.001$, and duration discrimination, $\rho$ = .95, 95% CI = [.72, .99], $p <.001$. Model fit is shown by the connected line segments in Fig. 4A,B. The shaded regions of Fig. 4 indicate 95% confidence intervals (CIs) obtained by iterative refitting of the model to 10,000 bootstrap-resampled datasets. Model CIs qualitatively reproduced the opposing effects of stimulus duration on duration and brightness discrimination across participants, underscoring the robustness of this perceptual reciprocity. Parameter estimates and model fit are reported in Table 1.

### Uncertainty principle depends on intensity

Experiment 2 examined how stimulus luminance affects perceptual sensitivity to both brightness and duration. Twenty participants viewed pairs of visual flashes in which the standard stimulus varied in luminance from 0.04 to 3.63 cd/m$^2$, while the standard duration remained fixed at 240 ms. In two separate sessions, they judged which of two flashes appeared brighter or longer.

As luminance increased, participants became more sensitive to stimulus duration: duration discrimination thresholds decreased from 76 ms to 58 ms across the luminance range, $F(4.21,\ 79.99) = 3.61$, $p =.008$, $\eta _G^2 =.06$, $\epsilon =.60$. In comparison, brightness discrimination performance declined systematically, with DLs rising from 0.01 to 1.2 cd/m$^2$, $F(1.39,\ 26.41) = 103.86$, $p <.001$, $\eta _G^2 =.80$, $\epsilon =.20$. Model predictions again closely tracked the behavioral threshold data for both brightness discrimination, $\rho$ = .999, 95% CI = [.997, .9999], $p <.001$, and duration discrimination, $\rho$ = .93, 95% CI = [.67, .99], $p <.001$. Bootstrapped 95% model CIs confirmed the stability of both effects qualitatively (see shaded regions in Fig. 4C,D).

Together, Experiments 1 and 2 reveal a reciprocal trade-off in perceptual resolution: as sensitivity to luminance increases, sensitivity to duration decreases, and vice versa. This empirical pattern aligns with theoretical predictions based on population-spike-count statistics, in which stochastic variability limits the joint precision of temporal and luminance encoding. We argue that the time–intensity uncertainty principle reflects a fundamental constraint on visual cognition.

## General discussion

The opposing effects of stimulus duration on brightness and temporal resolution observed in Experiment 1 align with long-standing principles of visual psychophysics. Increasing stimulus duration improved sensitivity to luminance contrast, consistent with Bloch’s law, which holds that luminance information is integrated over time to enhance detection and discrimination (Gorea & Tyler, 1986; Barlow, 1958; Legge, 1978; Graham & Kemp, 1938; Duysens et al., 1991). Conversely, temporal resolution declined with longer durations, mirroring the general trend expected based on Weber’s law for time perception, according to which duration discrimination thresholds scale proportionally with interval length (Getty, 1975; Wearden & Lejeune, 2008; Rammsayer et al., 2015; Heinrich et al., 2020; Church et al., 1976). Thus, as more sensory evidence accumulates over time, brightness discrimination improves, but the ability to resolve differences in duration degrades. We argue that these two classic laws share a common origin: a stochastic constraint imposed by gain-modulated Poisson population spike statistics in early visual cortex. Rather than reflecting independent mechanisms, Bloch’s law for temporal summation and Weber-like scaling of duration thresholds may emerge as complementary outcomes of the same spike-based uncertainty structure in the neural code, with gains in sensitivity to one dimension emerging only at the expense of the other.

Experiment 2 revealed a striking reciprocity in how stimulus luminance shapes resolution for perceived brightness versus perceived duration. Brightness DLs increased steeply with luminance, consistent with the general trend underlying Weber’s law for intensity discrimination (Bouman, 1952; Cornsweet & Teller, 1965; Scholtyssek et al., 2008). Conversely, duration DLs decreased as luminance rose, indicating improved temporal acuity. This luminance-dependent gain in temporal acuity parallels findings from psychoacoustics, where louder sounds yield finer timing judgments (Creelman, 1962; Henry, 1948; Sinnott et al., 1987). Although early visual studies reported mixed results for intensity effects on duration perception (Allan & Kristofferson, 1974), more recent work has shown that high-contrast stimuli steepen psychometric function slopes for duration discrimination (Skylark et al., 2010). Our results demonstrate that such improvements in temporal sensitivity are systematically accompanied by a loss of brightness sensitivity: a reciprocity well captured by the modulated Poisson model with a nonlinear transducer.

**Fig. 5:**
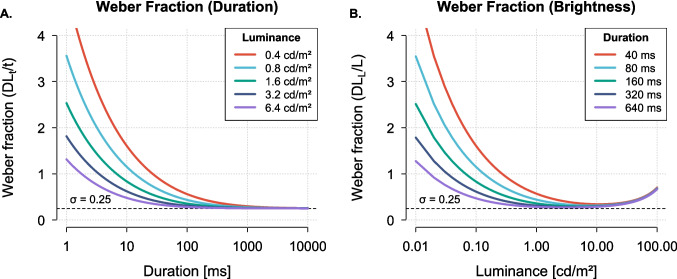
(**A**) Weber fractions for duration discrimination ($\textrm{DL}_t/t$) as a function of stimulus duration, shown for five luminance levels (0.4, 0.8, 1.6, 3.2, and 6.4 cd/m$^{2}$). Fractions decrease toward an asymptotic limit for long durations. (**B**) Weber fractions for brightness discrimination ($\textrm{DL}_L/L$) as a function of stimulus luminance, shown for five viewing times (40, 80, 160, 320, and 640 ms). Fractions initially decrease toward Weber’s law (roughly constant threshold) but rise again at high intensities due to response saturation. Note the effects of luminance integration (panel A) and temporal summation (panel B). Both panels assume $\lambda _{\text {max}}=10000$, $n=1$, $K=100$, and $\sigma ^2=0.0625$

Thus, the model explains the Weber-like trends across time and intensity. However, Weber’s law in its strict, idealized form (a constant Weber fraction) does not hold precisely for either brightness or duration. Instead, both attributes exhibit systematic deviations so that relative sensitivity improves at moderate stimulus levels before asymptoting: the “near-miss to Weber’s law” in intensity perception and the “generalized Weber function” in time perception (McGill & Goldberg, 1968; Treisman, 1964; Killeen & Weiss, 1987; Getty, 1975). The modulated Poisson model accounts for this nuanced pattern through its core stochastic structure. In a simple Poisson model, the Weber fraction follows an inverse square-root decay (Eq. B1), predicting ever-improving relative sensitivity. The introduction of gain variability counteracts this, causing the fraction to saturate and producing the observed near-miss/generalized Weber function (Ulrich et al., 2023; Zhou et al., 2024; Goris et al., 2018; Treisman, 1964). Figure 5 shows idealized Weber fractions for time ($\textrm{DL}_t/t$) and intensity ($\textrm{DL}_L/L$), generated with representative parameters estimated from our experiments and interpolated across behaviorally relevant stimulus ranges. As shown, gain-modulated Poisson statistics predict that both fractions should gradually decline toward an asymptotic bound determined by the gain variability ($\sigma$). The Naka–Rushton transducer causes $\textrm{DL}_L/L$ to rise again at high intensities due to response saturation eventually.

Although luminance and duration were each manipulated across a 2 $\log _{10}$ unit range, their effects on discrimination thresholds were asymmetric. In Experiment 1, brightness DLs fell by approximately 48% across the duration range, whereas in Experiment 2, duration DLs decreased by only 24% across the luminance range. This disparity reflects the nonlinear response of the underlying Naka–Rushton transducer: quasi-linear at low luminance, but compressive at moderate-to-high intensities. As a result, increasing stimulus duration drives steep gains in brightness sensitivity, whereas equivalent changes in luminance yield more modest improvements in temporal sensitivity. Also notably, the standard stimulus duration in Experiment 2 was fixed at 240 ms (a midrange value for this task), which may have further constrained the extent to which luminance could modulate temporal resolution. As shown in Fig. 3B, luminance effects on temporal sensitivity should be most pronounced at shorter durations, but saturate markedly for long-duration visual stimuli.

The fitted Naka–Rushton exponents *n* falls well within the range expected for population responses rather than single neurons. While single-unit contrast–response functions in V1 typically exhibit exponents around 2–3 (Sclar et al., 1990), population measures such as contrast-dependent V1 BOLD response consistently yield substantially shallower slopes closely matching our estimates (around 1; Boynton et al., 1996; Foster and Ling, 2022). By comparison, the fitted semisaturation constants *K* lie well above the physical luminance range tested, and cannot be interpreted as a biologically plausible response midpoint. We also implemented a fitting routine with custom constraints on the saturation constant, available as a supplementary analysis via our OSF depository. An upper roof of $K=10$ caused the model–data correlation for the brightness thresholds in Experiment 1 to drop from .99 to .87 but had a negligible influence on all other datapoints.

We acknowledge that our estimated saturation constant does not fall within a plausible range and can be interpreted only weakly in relation to the underlying biology. This mismatch might reflect some simplifying assumptions inherent to the model, as briefly discussed next. We also performed a joint fitting procedure that constrained model parameters to be identical across both experiments. While this only slightly reduced model–data correlations (all $\rho \ge 92$; see OSF depository), we opted for separate fits as our primary analysis because the two experiments involved different participant samples and experimental stimuli.

Our model makes several simplifying assumptions: All neurons follow monotonic Naka–Rushton transducers, omitting supersaturating cells with non-monotonic contrast tuning (Peirce, 2007; May & Zhaoping, 2011), and share identical parameters, ignoring heterogeneity in tuning shape and location that varies with spatial frequency selectivity and pathway division (Enroth-Cugell & Robson, 1966; Albrecht & Hamilton, 1982; Sclar et al., 1990). Gain fluctuations are modeled as trial-wise multiplicative noise, disregarding the fine temporal structure of gain fluctuations as observed in sensory cortex and captured by a recent continuous-time extension of the modulated Poisson model (Rupasinghe et al., 2025). Temporal adaptation and transient response dynamics are similarly neglected (Albrecht et al., 2002; Wong, 2021) and decoding is based on total spike count rather than more principled optimal-decoding strategies (Chirimuuta & Tolhurst, 2005; May & Solomon, 2015a; Chirimuuta et al., 2003; Clatworthy et al., 2003). Finally, we assume that the gain variance is stimulus-independent, which might be at odds with neurophysiological data (Festa et al., 2021; Goris et al., 2024; Hénaff et al., 2020). While our assumptions support tractability and preserve predictive clarity, they naturally limit mechanistic detail and completeness. Incorporating temporal dynamics, stimulus-dependent gain, or tuning heterogeneity could enrich future work.

The proposed time–intensity uncertainty principle offers a single computational framework for sensory resolution across multiple domains of visual processing. Classic psychophysical laws for time and brightness perception emerge here not from independent mechanisms, but from a shared stochastic constraint in visual coding. In this regard, the present work adheres to the broader scientific ideal of unification, whereby distinct laws or phenomena are integrated into a cohesive explanatory framework (Friedman, 1974). Notably, Zhou et al. (2024) recently showed that Weber’s law for perceptual sensitivity and Stevens’s (1961) power law for perceived magnitude (two cornerstones of behavioral psychophysics) naturally co-emerge from a modulated Poisson model with a nonlinear transducer, emerging asymptotically when response variability scales proportionally with mean activity while systematically deviating at lower stimulus levels. Zhou et al. (2024) and the present work both suggest that seemingly disparate psychophysical laws may arise from common stochastic limits in neural coding rather than independent mechanisms. This integrative approach suggests a possible path toward a unified theory of psychophysical behavior grounded in plausible models of neural information processing in the sensory cortex.

Nonetheless, it is worth noting that the observed trade-off could, in principle, stem from separate mechanisms. For example, duration discrimination could rely on a separate Poisson central clock (Treisman, 1963) which times the duration between transient onset and offset responses. If such a clock were similarly perturbed by fluctuating gain or some other additional variance source, it would yield largely indistinguishable behavioral predictions (Ulrich et al., 2023). Alternatively, brightness and duration might be processed in distinct visual areas (e.g., V1 versus extrastriate cortex). Given the prevalence of nonlinear transducers (Avidan et al., 2002; Yan et al., 2014) and overdispersed spike statistics (Goris et al., 2014; Hénaff et al., 2020) across visual regions, this dual-mechanism account could also explain the observed behavioral patterns. Although a single-mechanism model based on a shared V1 locus offers a parsimonious explanation, distinguishing among the alternatives will require additional empirical tests. Tentative support for the single-mechanism view comes from a PET-imaging study by Maquet et al. (1996) who found indistinguishable patterns of whole-brain activity in a temporal generalization task and a brightness generalization task, indicating that visual duration and brightness are represented within the same cortical networks.

In summary, we showed that longer visual stimuli enhance contrast sensitivity but impair temporal resolution, while brighter stimuli improve temporal acuity at the expense of contrast discrimination. This reciprocal pattern mirrors the ubiquitous uncertainty constraints in physics and engineering, such as the Gabor limit in communication theory, which states that signals cannot be both brief and spectrally precise. By analogy, we propose that the visual system cannot encode duration and intensity with arbitrarily high precision. Crucially, this time–intensity uncertainty principle does not depend on the specific nonlinear transducer or gain model assumed here, but arises generically if duration and intensity are inferred from a shared, Poisson-like spike code, with model details shaping the precise form of the trade-off rather than its existence. Finally, because the underlying logic reflects fundamental aspects of neural information processing, we speculate that similar trade-offs should arise across other sensory modalities, including audition and touch.

## Data Availability

Raw data are available at https://osf.io/zt6jw.

## Appendix

### Mathematical derivations

Starting from the negative binomial mass function in Eq. 2, define

A1 $$\begin{aligned} \mu =\textrm{E}[\textbf{N}]=\frac{\lambda _{\text {max}} L^n t}{L^n + K^n} \end{aligned}$$

as shorthand for the mean population spike count. The Weber fraction W is obtained from the coefficient of variation (Ulrich et al., 2023). Thus,

A2 $$\begin{aligned} \text {W}&= \frac{\sqrt{\text {Var}[\textbf{N}]}}{\mu } = \frac{\sqrt{\mu + \sigma ^2 \mu ^2}}{\mu } = \sqrt{\frac{1}{\mu } + \sigma ^2}\nonumber \\&= \sqrt{\frac{L^n + K^n}{\lambda _{\text {max}} L^n t} + \sigma ^2}. \end{aligned}$$

From Eq. A2, the duration threshold $\textrm{DL}_t = \text {W} \cdot t$ in Eq. 4 follows directly.

The expression for the luminance threshold in Eq. 5 requires relating a just-noticeable change in spike rate ($\Delta \lambda$) to the corresponding change in luminance ($\text {DL}_L$). We know that the smallest noticeable rate change is

A3 $$\begin{aligned} \Delta \lambda = \text {W} \cdot \lambda (L). \end{aligned}$$

The luminance threshold can therefore be obtained by linearizing the relationship between luminance and firing rate using the local slope of the transducer. A just-noticeable change in spike rate $\Delta \lambda$ corresponds to a change in luminance given by

A4 $$\begin{aligned} \textrm{DL}_L \approx \frac{\Delta \lambda }{\lambda '(L)} = \text {W} \cdot \lambda (L)\cdot \left( \frac{\partial \lambda (L)}{\partial L}\right) ^{-1}. \end{aligned}$$

This expression follows from a first-order Taylor expansion of the inverse transducer $L(\lambda )$ around the operating point $\lambda (L)$,

A5 $$\begin{aligned} L(\lambda + \Delta \lambda ) \approx L(\lambda ) + \frac{\partial L}{\partial \lambda } \Delta \lambda \end{aligned}$$

where $\partial L / \partial \lambda =[\lambda '(L)]^{-1}$. Inserting the expression for the Naka–Rushton transducer function and simplifying yields

A6 $$\begin{aligned} \lambda (L) \cdot \left( \frac{\partial \lambda (L)}{\partial L}\right) ^{-1}&= \frac{\lambda _{\text {max}} L^n}{L^n + K^n} \cdot \frac{(L^n + K^n)^2}{n \lambda _{\text {max}} K^n L^{n-1}}\nonumber \\&= \frac{L (L^n +K^n)}{nK^n}. \end{aligned}$$

Substituting Eq. A2 then gives the linearized expression

A7 $$\begin{aligned} \text {DL}_L&\approx \text {W} \cdot \frac{L (L^n +K^n)}{nK^{n}}=\frac{L(L^n+K^n)\sqrt{\frac{(L^n +K^n)}{\lambda _{\text {max}}tL^{n}}+\sigma ^2}}{nK^n}. \end{aligned}$$

This approximation highlights some basic dependencies, such as the inverse dependence on stimulus duration *t*.

An exact expression for $\text {DL}_L$ similarly follows from the smallest noticeable change in population rate $\Delta \lambda =\text {W} \cdot \lambda (L)$. The corresponding population rates at upper (+) and lower (−) thresholds are

A8 $$\begin{aligned} \lambda ^\pm _{\text {th}} = \lambda (L) \pm \Delta \lambda = (1 \pm \text {W}) \cdot \lambda (L). \end{aligned}$$

Using the Naka–Rushton inverse function

A9 $$\begin{aligned} L(\lambda ) = \left( \frac{\lambda K^n}{\lambda _{\text {max}} - \lambda }\right) ^{1/n}, \end{aligned}$$

the luminance at the upper and lower thresholds is

A10 $$\begin{aligned} L^\pm _{\text {th}}&= \left( \frac{\lambda ^\pm _{\text {th}} K^n}{\lambda _{\text {max}} - \lambda ^\pm _{\text {th}}}\right) ^{1/n} = \left( \frac{\lambda (L)(1 \pm \text {W})K^n}{\lambda _{\text {max}} - \lambda (L)(1 \pm \text {W})}\right) ^{1/n}. \end{aligned}$$

Thus, the exact two-sided $\text {DL}_L$ is

A11 $$\begin{aligned} \text {DL}_L = \frac{L^+_{\text {th}} - L^-_{\text {th}}}{2}. \end{aligned}$$

Equation A11 follows the standard operationalization of a DL as half the distance between its upper and lower estimates (Duncan Luce & Galanter, 1963). Across the range of stimulus values and parameter estimates considered here, the approximate (Eq. A7) and exact (Eq. A11) methods agree to within 1%. Code reproducing this agreement is available via https://osf.io/zt6jw. All algebraic manipulations and closed-form expressions were independently verified using symbolic software (WolframAlpha).

## Code availability

Analysis scripts are available at https://osf.io/zt6jw.

## References

[CR1] Albrecht, D. G., et al. (2002). Visual cortex neurons of monkeys and cats: temporal dynamics of the contrast response function. *Journal of Neurophysiology,**88*, 888–913. 10.1152/jn.2002.88.2.888 12163540 10.1152/jn.2002.88.2.888

[CR2] Albrecht, D. G., & Hamilton, D. B. (1982). Striate cortex of monkey and cat: contrast response function. *Journal of Neurophysiology,**48*, 217–237. 10.1152/jn.1982.48.1.217 7119846 10.1152/jn.1982.48.1.217

[CR3] Allan, L. G., & Kristofferson, A. B. (1974). Psychophysical theories of duration discrimination. *Perception & Psychophysics,**16*, 26–34. 10.3758/BF03203244

[CR4] Avidan, G., et al. (2002). Contrast sensitivity in human visual areas and its relationship to object recognition. *Journal of Neurophysiology,**87*, 3102–3116. 10.1152/jn.2002.87.6.3102 12037211 10.1152/jn.2002.87.6.3102

[CR5] Barlow, H. B. (1958). Temporal and spatial summation in human vision at different background intensities. *Journal of Physiology,**141*, 337–350. 10.1113/jphysiol.1958.sp005978 13539843 10.1113/jphysiol.1958.sp005978 PMC1358805

[CR6] Billock, V. A., & Tsou, B. H. (2011). To honor Fechner and obey Stevens: Relationships between psychophysical and neural nonlinearities. *Psychological Bulletin,**137*, 1–18. 10.1037/a0021394 21219055 10.1037/a0021394

[CR7] Bouman, M. A. (1952). Peripheral contrast thresholds for various and different wavelengths for adapting field and test stimulus. *Journal of the Optical Society of America,**42*, 820–831. 10.1364/JOSA.42.000820 13011671 10.1364/josa.42.000820

[CR8] Boynton, G. M., et al. (1996). Linear systems analysis of functional magnetic resonance imaging in human V1. *Journal of Neuroscience,**16*, 4207–4221. 10.1523/JNEUROSCI.16-13-04207.1996 8753882 10.1523/JNEUROSCI.16-13-04207.1996 PMC6579007

[CR9] Boynton, G. M., et al. (1999). Neuronal basis of contrast discrimination. *Vision Research,**39*, 257–269. 10.1016/s0042-6989(98)00113-8 10326134 10.1016/s0042-6989(98)00113-8

[CR10] Buhusi, C. V., & Meck, W. H. (2005). What makes us tick? Functional and neural mechanisms of interval timing. *Nature Reviews Neuroscience,**6*, 755–765. 10.1038/nrn1764 16163383 10.1038/nrn1764

[CR11] Chirimuuta, M., Clatworthy, P. L., & Tolhurst, D. J. (2003). Coding of the contrasts in natural images by visual cortex (V1) neurons: A Bayesian approach. *Journal of the Optical Society of America,**20*, 1253–1260. 10.1364/JOSAA.20.001253 12868631 10.1364/josaa.20.001253

[CR12] Chirimuuta, M., & Tolhurst, D. J. (2005). Does a Bayesian model of V1 contrast coding offer a neurophysiological account of human contrast discrimination? *Vision Research,**45*, 2943–2959. 10.1016/j.visres.2005.06.022 16081128 10.1016/j.visres.2005.06.022

[CR13] Church, R. M., Getty, D. J., & Lerner, N. D. (1976). Duration discrimination by rats. *Journal of Experimental Psychology: Animal Behavior Processes,**2*, 303–312. 10.1037/0097-7403.2.4.303 988110 10.1037//0097-7403.2.4.303

[CR14] Clatworthy, P. L., et al. (2003). Coding of the contrasts in natural images by populations of neurons in primary visual cortex (V1). *Vision Research,**43*, 1983–2001. 10.1016/S0042-6989(03)00277-3 12831760 10.1016/s0042-6989(03)00277-3

[CR15] Cornsweet, T. N., & Teller, D. Y. (1965). Relation of increment thresholds to brightness and luminance. *Journal of the Optical Society of America,**55*, 1303–1308. 10.1364/josa.55.001303 5888119 10.1364/josa.55.001303

[CR16] Coull, J. T., Cheng, R.-K., & Meck, W. H. (2011). Neuroanatomical and neurochemical substrates of timing. *Neuropsychopharmacology,**36*, 3–25. 10.1038/npp.2010.113 20668434 10.1038/npp.2010.113 PMC3055517

[CR17] Creelman, C. D. (1962). Human Discrimination of Auditory Duration. *Journal of the Acoustical Society of America,**34*, 582–593. 10.1121/1.1918172

[CR18] Diamond, J. S., & Copenhagen, D. R. (1995). The relationship between light-evoked synaptic excitation and spiking behaviour of salamander retinal ganglion cells. *Journal of Physiology,**487*, 711–725. 10.1113/jphysiol.1995.sp020912 8544133 10.1113/jphysiol.1995.sp020912 PMC1156657

[CR19] Duysens, J., Gulyas, B., & Maes, H. (1991). Temporal integration in cat visual cortex: A test of Bloch’s law. *Vision Research,**31*, 1517–1528. 10.1016/0042-6989(91)90129-s 1949621 10.1016/0042-6989(91)90129-s

[CR20] Ecker, A. S., et al. (2014). State dependence of noise correlations in macaque primary visual cortex. *Neuron,**82*, 235–248. 10.1016/j.neuron.2014.02.006 24698278 10.1016/j.neuron.2014.02.006 PMC3990250

[CR21] Enroth-Cugell, C., & Robson, J. G. (1966). The contrast sensitivity of retinal ganglion cells of the cat. *Journal of Physiology,**187*, 517–552. 10.1113/jphysiol.1966.sp008107 16783910 10.1113/jphysiol.1966.sp008107 PMC1395960

[CR22] Evans, L. S., Peachey, N. S., & Marchese, A. L. (1993). Comparison of three methods of estimating the parameters of the Naka-Rushton equation. *Documenta Ophthalmologica,**84*, 19–30. 10.1007/BF01203279 8223107 10.1007/BF01203279

[CR23] Festa, D., et al. (2021). Neuronal variability reflects probabilistic inference tuned to natural image statistics. *Nature Communications,**12*, 3635. 10.1038/s41467-021-23838-x 34131142 10.1038/s41467-021-23838-x PMC8206154

[CR24] Foster, J. J., & Ling, S. (2022). Feature-based attention multiplicatively scales the fMRI-BOLD contrast-response function. *Journal of Neuroscience,**42*, 6894–6906. 10.1523/JNEUROSCI.0513-22.2022 35868860 10.1523/JNEUROSCI.0513-22.2022 PMC9464014

[CR25] Friedman, M. (1974). Explanation and scientific understanding. *Journal of Philosophy,**71*, 5–19. 10.2307/2024924

[CR26] Gabor, D. (1946). Theory of communication. Part 1: The analysis of information. *Journal of the Institution of Electrical Engineers-part III: Radio and Communication Engineering,**93*, 429–441. 10.1049/ji-3-2.1946.0074

[CR27] Getty, D. J. (1975). Discrimination of short temporal intervals: A comparison of two models. *Perception & Psychophysics,**18*, 1–8. 10.3758/BF03199358

[CR28] Gibbon, J. (1992). Ubiquity of scalar timing with a Poisson clock. *Journal of Mathematical Psychology,**36*, 283–293. 10.1016/0022-2496(92)90041-5

[CR29] Gorea, A., & Tyler, C. (1986). New look at Bloch’s law for contrast. *Journal of the Optical Society of America A,**3*, 52–61. 10.1364/JOSAA.3.000052 10.1364/josaa.3.000052 3950792

[CR30] Goris, R. L. T., et al. (2024). Response sub-additivity and variability quenching in visual cortex. *Nature Reviews Neuroscience*, *25*, 237–252. 10.1038/s41583-024-00795-0 10.1038/s41583-024-00795-0 PMC11444047 38374462

[CR31] Goris, R. L. T., Movshon, J. A., & Simoncelli, E. P. (2014). Partitioning neuronal variability. *Nature Neuroscience*, *17*, 858–865. 10.1038/nn.3711 10.1038/nn.3711 PMC4135707 24777419

[CR32] Goris, R. L. T., et al. (2018). Slow gain fluctuations limit benefits of temporal integration in visual cortex. *Journal of Vision,**18*, 8. 10.1167/18.8.8 30140890 10.1167/18.8.8 PMC6107324

[CR33] Graham, C. H., & Kemp, E. H. (1938). Brightness discrimination as a function of the duration of the increment in intensity. *Journal of General Physiology,**21*, 635–650. 10.1085/jgp.21.5.635 19873072 10.1085/jgp.21.5.635 PMC2141958

[CR34] Green, D. M., & Luce, R. D. (1974). Counting and timing mechanisms in auditory discrimination and reaction time. In D. H. Krantz, et al. (eds.), *Contemporary Developments in Mathematical Psychology: Vol. II*, San Francisco: W.H. Freeman and Company, (pp. 372–431).

[CR35] Heinrich, T., Ravignani, A., & Hanke, F. D. (2020). Visual timing abilities of a harbour seal (Phoca vitulina) and a South African fur seal (Arctocephalus pusillus pusillus) for sub-and supra-second time intervals. *Animal Cognition,**23*, 851–859. 10.1007/s10071-020-01390-3 32388781 10.1007/s10071-020-01390-3 PMC7415748

[CR36] Heisenberg, W. (1958/2006). *Physik und Philosophie*. 7th ed. Quotation from (p. 149) f., translated by the authors. Stuttgart: S. Hirzel Verlag.

[CR37] Heisenberg, W. (1927). Über den anschaulichen Inhalt der quantentheoretischen Kinematik und Mechanik [The actual content of quantum theoretical kinematics and mechanics]. *Zeitschrift für Physik,**43*, 172–198. 10.1007/BF01397280

[CR38] Hénaff, O. J., et al. (2020). Representation of visual uncertainty through neural gain variability. *Nature Communications,**11*, 2513. 10.1038/s41467-020-15533-0 32427825 10.1038/s41467-020-15533-0 PMC7237668

[CR39] Henry, F. M. (1948). Discrimination of the duration of a sound. *Journal of Experimental Psychology,**38*, 734–743. 10.1037/h0058552 18893188 10.1037/h0058552

[CR40] Kaernbach, C. (1991). Simple adaptive testing with the weighted up-down method. *Perception & Psychophysics,**49*, 227–229. 10.3758/BF03214307 2011460 10.3758/bf03214307

[CR41] Killeen, P., & Weiss, N. (1987). Optimal timing and the weber function. *Psychological Review,**94*, 455–468. 10.1037/0033-295X.94.4.455 3317471

[CR42] Legge, G. E. (1978). Sustained and transient mechanisms in human vision: Temporal and spatial properties. *Vision Research,**18*, 69–81. 10.1016/0042-6989(78)90079-2 664278 10.1016/0042-6989(78)90079-2

[CR43] Luce, R. D., & Galanter, E. (1963). Discrimination. In Luce, R.D., Bush, R.R., & Eugene, G. (eds.), *Handbook of Mathematical Psychology*. Wiley & Sons., (pp. 191–243).

[CR44] Maquet, P., et al. (1996). Brain activation induced by estimation of duration: A PET study. *NeuroImage,**3*, 119–126. 10.1006/nimg.1996.0014 9345483 10.1006/nimg.1996.0014

[CR45] May, K. A., & Solomon, J. A. (2015a). Connecting psychophysical performance to neuronal response properties I: Discrimination of suprathreshold stimuli. *Journal of Vision*, *15*, 8. 10.1167/15.6.8 10.1167/15.6.8 26024455

[CR46] May, K. A., & Solomon, J. A. (2015b). Connecting psychophysical performance to neuronal response properties II: Contrast decoding and detection. *Journal of Vision*, *15*, 9. 10.1167/15.6.9 10.1167/15.6.9 26024456

[CR47] May, K. A., & Zhaoping, L. (2011). Exploring the roles of saturating and supersaturating contrast-response functions in conjunction detection and contrast coding. *Journal of Vision,**11*, 11. 10.1167/11.9.11 21862639 10.1167/11.9.11

[CR48] McGill, W. J. (1967). Neural counting mechanisms and energy detection in audition. *Journal of Mathematical Psychology,**4*, 351–376. 10.1016/0022-2496(67)90030-2

[CR49] McGill, W. J., & Goldberg, J. (1968). A study of the near-miss involving Weber’s law and pure-tone intensity discrimination. *Perception & Psychophysics,**4*, 105–109. 10.3758/BF03209518

[CR50] Morey, R. (2008). Confidence Intervals from Normalized Data: A Correction to Cousineau (2005). *Tutorials in Quantitative Methods for Psychology,**4*, 61–64. 10.20982/tqmp.04.2.p061

[CR51] Paton, J. J., & Buonomano, D. V. (2018). The neural basis of timing: distributed mechanisms for diverse functions. *Neuron,**98*, 687–705. https://doi.org/j.neuron.2018.03.045 10.1016/j.neuron.2018.03.045 PMC5962026 29772201

[CR52] Peirce, J. W. (2007). The potential importance of saturating and supersaturating contrast response functions in visual cortex. *Journal of Vision,**7*, 13. 10.1167/7.6.13 17685796 10.1167/7.6.13 PMC2082665

[CR53] Peirce, J. W., et al. (2019). PsychoPy2: Experiments in behavior made easy. *Behavior Research Methods,**51*, 195–203. 10.3758/s13428-018-01193-y 30734206 10.3758/s13428-018-01193-y PMC6420413

[CR54] Plant, R. R., Hammond, N., & Turner, G. (2004). Self-validating presentation and response timing in cognitive paradigms: How and why? *Behavior Research Methods, Instruments, & Computers,**36*, 291–303. 10.3758/BF03195575 10.3758/bf03195575 15354695

[CR55] Raab, D. H., & Goldberg, I. (1975). Auditory intensity discrimination with bursts of reproducible noise. *Journal of the Acoustical Society of America,**57*, 437–447. 10.1121/1.380467 1117097 10.1121/1.380467

[CR56] Rabinowitz, N. C., et al. (2015). Attention stabilizes the shared gain of V4 populations. *eLife,**4*, e08998. 10.7554/eLife.08998 26523390 10.7554/eLife.08998 PMC4758958

[CR57] Rammsayer, T., Borter, N., & Troche, S. (2015). Visual-auditory differences in duration discrimination of intervals in the subsecond and second range. *Frontiers in Psychology,**6*, 1626. 10.3389/fpsyg.2015.01626 26579013 10.3389/fpsyg.2015.01626 PMC4620148

[CR58] Rammsayer, T., & Ulrich, R. (2001). Counting models of temporal discrimination. *Psychonomic Bulletin & Review,**8*, 270–277. 10.3758/BF03196161 11495114 10.3758/bf03196161

[CR59] Rupasinghe, A., Charles, A. S., & Pillow, J. W. (2025). Continuous partitioning of neuronal variability. In: bioRxiv. Preprint. 10.1101/2025.07.23.666404.

[CR60] Scholtyssek, C., Kelber, A., & Dehnhardt, G. (2008). Brightness discrimination in the harbor seal (Phoca vitulina). *Vision Research,**48*, 96–103. 10.1016/j.visres.2007.10.012 18078667 10.1016/j.visres.2007.10.012

[CR61] Sclar, G., Maunsell, J. H. R., & Lennie, P. (1990). Coding of image contrast in central visual pathways of the macaque monkey. *Vision Research,**30*, 1–10. 10.1016/0042-6989(90)90123-3 10.1016/0042-6989(90)90123-3 2321355

[CR62] Siebert, W. M. (1965). Some implications of the stochastic behavior of primary auditory neurons. *Kybernetik,**2*, 206–215. 10.1007/BF00306416 5839007 10.1007/BF00306416

[CR63] Simen, P., et al. (2013). Timescale invariance in the pacemaker-accumulator family of timing models. *Timing & Time Perception,**1*, 159–188. 10.1163/22134468-00002018

[CR64] Sinnott, J. M., Owren, M. J., & Petersen, M. R. (1987). Auditory duration discrimination in Old World monkeys *(Macaca, Cercopithecus)* and humans. *Journal of the Acoustical Society of America,**82*, 465–470. 10.1121/1.395447 10.1121/1.395447 3624651

[CR65] Skylark, W., Stewart, N., & Wearden, J. (2010). Stimulus intensity and the perception of duration. *Journal of Experimental Psychology: Human Perception and Performance,**37*, 303–313. 10.1037/a0019961 10.1037/a0019961 20731508

[CR66] Stevens, S. S. (1961). To Honor Fechner and Repeal His Law: A power function, not a log function, describes the operating characteristic of a sensory system. *Science,**133*, 80–86. 10.1126/science.133.3446.80 17769332 10.1126/science.133.3446.80

[CR67] Treisman, M. (1963). Temporal discrimination and the indifference interval: Implications for a model of the “internal clock.’’. *Psychological Monographs,**77*, 1–31. 10.1037/h0093864 5877542 10.1037/h0093864

[CR68] Treisman, M. (1964). Noise and Weber’s law: The discrimination of brightness and other dimensions. *Psychological Review,**71*, 314–30. 10.1037/h0042445 14183614 10.1037/h0042445

[CR69] Ulrich, R., Bausenhart, K., & Wearden, J. H. (2023). Weber’s law for timing and time perception: reconciling the poisson clock with Scalar Expectancy Theory (SET). *Timing & Time Perception,**11*, 167–197. 10.1163/22134468-bja10055

[CR70] Wearden, J., & Lejeune, H. (2008). Scalar Properties in Human Timing: Conformity and Violations. *Quarterly Journal of Experimental Psychology,**61*, 569–587. 10.1080/17470210701282576 10.1080/17470210701282576 18938276

[CR71] Wong, W. (2021). Consilience in the peripheral sensory adaptation response. *Frontiers in Human Neuroscience,**15*, 727551. 10.3389/fnhum.2021.727551 34744660 10.3389/fnhum.2021.727551 PMC8569822

[CR72] Yan, T., et al. (2014). Contrast response functions with wide-view stimuli in the human visual cortex. *Perception,**43*, 677–693. 10.1068/p7640 25223111 10.1068/p7640

[CR73] Zhou, J., Duong, L. R., & Simoncelli, E. P. (2024). A unified framework for perceived magnitude and discriminability of sensory stimuli. *Proceedings of the National Academy of Sciences,**121*, e2312293121. 10.1073/pnas.2312293121 10.1073/pnas.2312293121 PMC11194506 38857385

[CR74] Zwislocki, J. J., & Jordan, H. N. (1986). On the relations of intensity jnd’s to loudness and neural noise. *Journal of the Acoustical Society of America,**79*, 772–780. 10.1121/1.393467 3958318 10.1121/1.393467

